# Raman spectroscopy to discriminate laryngeal squamous cell carcinoma from non-cancerous surrounding tissue

**DOI:** 10.1007/s10103-023-03849-4

**Published:** 2023-08-25

**Authors:** Cornelia van Lanschot, Tom Bakker Schut, Elisa Barroso, Aniel Sewnaik, Jose Hardillo, Dominiek Monserez, Cees Meeuwis, Stijn Keereweer, Rob Baatenburg de Jong, Gerwin Puppels, Senada Koljenović

**Affiliations:** 1 Department of Otorhinolaryngology and Head and Neck Surgery, Erasmus MC, University Medical Center Rotterdam, PO Box 2040, 3000 CA Rotterdam, The Netherlands; 2 Center for Optical Diagnostics and Therapy, Department of Dermatology, Erasmus MC, University Medical Center Rotterdam, PO Box 2040, 3000 CA Rotterdam, The Netherlands; 3 Department of Oral and Maxillofacial Surgery, Erasmus MC, University Medical Center Rotterdam, PO Box 2040, 3000 CA Rotterdam, The Netherlands; 4 Department of Pathology, Erasmus MC, University Medical Center Rotterdam, PO Box 2040, 3000 CA Rotterdam, The Netherlands; 5 Department of Pathology, Antwerp University Hospital, 2650 Antwerp, Belgium; 6 Faculty of Medicine, University of Antwerp, 2610 Antwerp, Belgium

**Keywords:** Larynx, Squamous cell carcinoma, Raman spectroscopy, High wavenumber

## Abstract

As for many solid cancers, laryngeal cancer is treated surgically, and adequate resection margins are critical for survival. Raman spectroscopy has the capacity to accurately differentiate between cancer and non-cancerous tissue based on their molecular composition, which has been proven in previous work. The aim of this study is to investigate whether Raman spectroscopy can be used to discriminate laryngeal cancer from surrounding non-cancerous tissue. Patients surgically treated for laryngeal cancer were included. Raman mapping experiments were performed ex vivo on resection specimens and correlated to histopathology. Water concentration analysis and CH-stretching region analysis were performed in the high wavenumber range of 2500–4000 cm^−1^. Thirty-four mapping experiments on 22 resection specimens were used for analysis. Both laryngeal cancer and all non-cancerous tissue structures showed high water concentrations of around 75%. Discriminative information was only found to be present in the CH-stretching region of the Raman spectra of the larynx (discriminative power of 0.87). High wavenumber region Raman spectroscopy can discriminate laryngeal cancer from non-cancerous tissue structures. Contrary to the findings for oral cavity cancer, water concentration is not a discriminating factor for laryngeal cancer.

**Supplementary Information:**

The online version contains supplementary material available at 10.1007/s10103-023-03849-4.

## Introduction

Surgical treatment with adequate resection margins is an important prognostic parameter for patients with laryngeal squamous cell carcinoma (LSCC) [1–3]. van Lanschot et al. described a significantly worse survival for positive margins in LSCC compared to those with close and clear. Yet, avoiding positive margins is difficult, due to the anatomy of the larynx [4]. To guide the surgeon towards an adequate resection, intra-operative assessment with frozen section analysis is often performed [4, 5], especially in cases where preservation of speech and swallowing is possible [6, 7]. However, this method is time-consuming and prone to sampling error [4, 5]. A rapid and objective technology would be of added value for the intra-operative assessment of resection margins.

Various optical techniques have been investigated for this important clinical oncological need. These optical techniques have opened new perspectives because of their ease of use, high speed, and real-time and objective tissue characterization [8–11]. Narrow-band imaging (NBI), autofluorescence (AF), contact endoscopy (CE), optical coherence tomography (OCT), fluorescence imaging (FLI), and Raman spectroscopy are examples of these techniques. NBI, AF, CE, and OCT are established tools for early detection of laryngeal cancer but their intra-operative function is limited by the inability to image beyond the (sub-)mucosa. These techniques are only useful for mucosal margin assessment and unsuitable for deep margin assessment. Fluorescence imaging has the potential for assessment of deeper margins because of the possibility to image at considerable depths and tissues other than the mucosa. Unfortunately, research on FLI for margin assessment is sparse. Raman spectroscopy has important advantages allowing for margin assessment in the deep margin planes. Raman spectroscopy is a technique based on the inelastic scattering of light in tissue and is able to characterize all different tissue types [11, 12]. Many studies have shown the potential of Raman spectroscopy to discriminate between cancer and non-cancerous tissue [11]. Increased accuracy for cancer diagnosis with Raman spectroscopy–based biopsy is reported with sensitivities between 73 and 100% and specificities of 66–100% [11].

Although promising, for laryngeal tissue, there are only a few feasibility studies performed on cancer detection with Raman spectroscopy. Different research groups determined that Raman spectra can be obtained rapidly and can reveal differences between non-cancerous tissue and LSCC [13–17]. These findings are based on the global spectral differences between cancerous and non-cancerous tissue, and the exact differences in discrimination between tumors and different surrounding tissue structures like connective tissue, gland, cartilage, muscle, and necrotic tissue were not analyzed.

In the literature mostly the fingerprint region is used, in this study it is chosen to use the high wavenumber region (2500–4000 cm^−1^) because of the stronger Raman signals and reduced fluorescence background, and because it enables in vivo application using simple single fiber optical probes [18].

In previous work, it was demonstrated that high wavenumber Raman spectroscopy can discriminate oral cavity squamous cell carcinoma (OCSCC) from surrounding non-cancerous tissue with 99% sensitivity and 92% specificity based on water concentration. Based on this information, the tumor border could be determined, with a decrease in concentration from 76% inside the tumor to 54% at 5 mm from the tumor border [19]. The aim of this study was to investigate the spectral differences between tumors and the different tissue structures and to determine whether high wavenumber Raman spectroscopy can be used to discriminate laryngeal cancer from surrounding non-cancerous tissue.

## Material and methods

### Medical ethical approval

The study was approved by the Medical Ethics Committee of the Erasmus MC, University Medical Center Rotterdam (MEC-2013–345). Informed consent was obtained from all patients before treatment.

### Study population

Patients surgically treated (e.g., laryngectomy) between December 2015 and January 2019 for LSCC were included in this study. The experiments were performed ex vivo on resection specimens of the patients directly after surgery.

### Tissue sampling

The time for experiments was limited to 60 min for optimal preservation of the tissue for the final pathological evaluation. After surgery, the specimen was brought to the cutting room of the pathology department. A dedicated head and neck pathologist (author) and the surgeon performed an intra-operative assessment of the resection margins, as described by Author et al. [20]. After this assessment, a tissue section was cut from the specimen for the Raman experiments (hereafter referred to as Raman section), without interfering with the standard pathological evaluation of the specimen. All steps handling the resection specimen were recorded. The Raman section was rinsed from blood with physiological salt solution (0.9% NaCl) and patted dry. Afterwards, the tissue section was inserted in a closed cartridge of which the upper side consists of a fused silica window. The fused silica window was in contact with the tissue section. The maximum tissue area that could be scanned was determined by the size of the cartridge which was 3 × 3cm^2^. If possible, both sides of the Raman section were scanned. After the experiment, the Raman section was added to the main specimen for fixation in formalin, for routine histopathologic evaluation.

### Raman instrumentation and experiments

A confocal Raman microscope was used to perform experiments. The setup is described in detail in an earlier study [19]. The equipment contains a 671 nm laser (CL671-150-SO, CrystaLaser), a charge-coupled device (CCD) camera fitted with a back-illuminated deep depletion CDD-chip (Andor iDus 401, DU401A BR-DD, Andor Technology Ltd.), and a multichannel Raman Module (HPRM 2500, RiverD International B.V). The Raman Module was coupled to the microscope (Leica DM RXA2, Leica Microsystems Wetzlar GmbH) and a computer-controlled sample stage (Leica DM STC). The cartridge, with the Raman section, was fixed on the stage and the surface of the tissue was mapped in a grid. The step size of the grid varied between 250 µm × 250 µm and 1000 µm × 1000 µm depending on the size of the tissue section and the maximum time to perform the experiment. Laser light (80mW) was focused through the microscope objective (0.4 numerical apertures) with a 1.1 mm working distance (NPLAN 11566026, Leica Microsystems B.V.). The laser was focused in the tissue, 50–70 µm below the fused silica window. The signal acquisition time per spectrum was 1 s. Spectral information was collected in the high wavenumber range of 2500–4000 cm^−1^ with a resolution < 5 cm^−1^ and a depth resolution of 40 µm.

### Calibration and processing data

Software for calibration and processing of the collected spectra was developed with MATLAB (R2017b). Spectra were calibrated on the relative wavenumber axis and corrected for wavelength-dependent detection efficiency of the setup, according to instructions of the spectrometer manufacturer (RiverD International B.V.). Preprocessing of the spectra was performed by removal of cosmic ray events and subtraction of signal background generated in the optical path of the setup [21]. The background signal, caused by tissue autofluorescence, was estimated as a 3rd-order polynomial and subtracted from the preprocessed spectra [19]. For all spectra, the average intensity over the range of 2700–3100 cm^−1^ was used as a measure of signal intensity. The lowest quality spectra (with an average intensity < 5% of the overall average intensity) were excluded from the analysis.

### Histopathology

Histopathologic evaluation of the Raman section was performed by a head and neck expert pathologist (author) on a hematoxylin and eosin (HE)-stained section. The HE-stained section was digitized, and the pathologist delineated different tissue types (e.g., tumor, connective tissue, gland, cartilage, muscle, necrotic tissue) on the digitized section.

### Data analysis

Data analysis was performed in MATLAB; R2017b. Data analysis consisted of (1) analysis of the water concentration, (2) analysis of the CH-stretching region by performing principal component analysis (PCA), and linear discriminant analysis (LDA).

#### Water concentration analysis

The water concentration in the Raman section was calculated for each measurement point according to the method developed by Caspers et al. [19]. Spectra with water percentages > 88% were considered outliers and discarded from the analysis. Raman maps were created by plotting the water concentration as a 2D map with color codes representing the range in water concentration. The Raman map was averaged (convoluted with a 3 × 3 average filter) to obtain a representative water concentration (reducing noise in the image), and better visualization of the difference in water concentration between tumor and non-cancerous tissue [22]. The delineated images of the tissue sections were projected over the corresponding Raman maps. For each Raman map, areas were selected with unambiguous histological annotation. The Raman data from these areas were used for data analysis. The precision of the annotation was limited by the resolution of the Raman map (pixel size varying from 250 µm × 250 µm to 1000 µm × 1000 µm). Afterwards, the water concentrations were separated into two groups: water concentrations from the non-cancerous tissue and water concentrations from the tumor. A Wilcoxon rank sum test was used to test whether the water concentration distributions were significantly different at the 0.05 confidence level. The discriminatory power for tissue classification based on water concentration was determined by measuring the area under the ROC curve (receiver operating characteristic curve).

#### CH-stretching region analysis

For analysis of the CH-stretching Raman signal, the spectral region between 2800 and 3100 cm^−1^ was used, which is independent of, and complementary to, the water concentration analysis. All spectra were scaled using an extended multiplicative scatter correction (EMSC) procedure (using water as a spectral interferent) [23] to eliminate spectral interference of varying water contributions. This method has been used in earlier studies and ensures the CH-stretching region analysis is independent of the water signal [24]. For each Raman map, a color map based on a PCA on the CH-stretching data was made. The scores on the first 3 (most significant) principal components (PCs) were used as input data for the red, blue, and green channels of the color map. In this way, the most important signal variance in the CH-stretching region is displayed as an image and can be compared to histology. PCA on the whole dataset was performed to reduce the dimensionality of data before LDA modeling. The spectra were first filtered with a Savitzky-Golay filter (order 3, window size of 11 points) to reduce the influence of noise on the PCA result. To separate tumor spectra from non-cancerous spectra, LDA was used to find the direction in PCA space that maximizes the ratio between the inter-group and intra-group variances [25, 26]. The scores on the first (most significant) PCs were selected as input parameters for the LDA. The optimal number of PCs for the PCA-LDA model was determined by leave-one-map-out validation on a model data set with the spectra of 25 maps from 17 patients. The PCA-LDA model was validated with an independent test data set with the spectra of 9 maps from 5 patients. ROC curve analysis was used to determine the discriminative power of the LDA analysis.

## Results

Forty-seven ex vivo Raman experiments were performed on laryngectomy specimens from 27 patients.

For seven experiments, the registration of the measured region was not reliable, and therefore correlation with histopathology was not performed. Six experiments were excluded because the general spectral quality was insufficient. Finally, thirty-four experiments were included for further analysis.

**Fig. 1:**
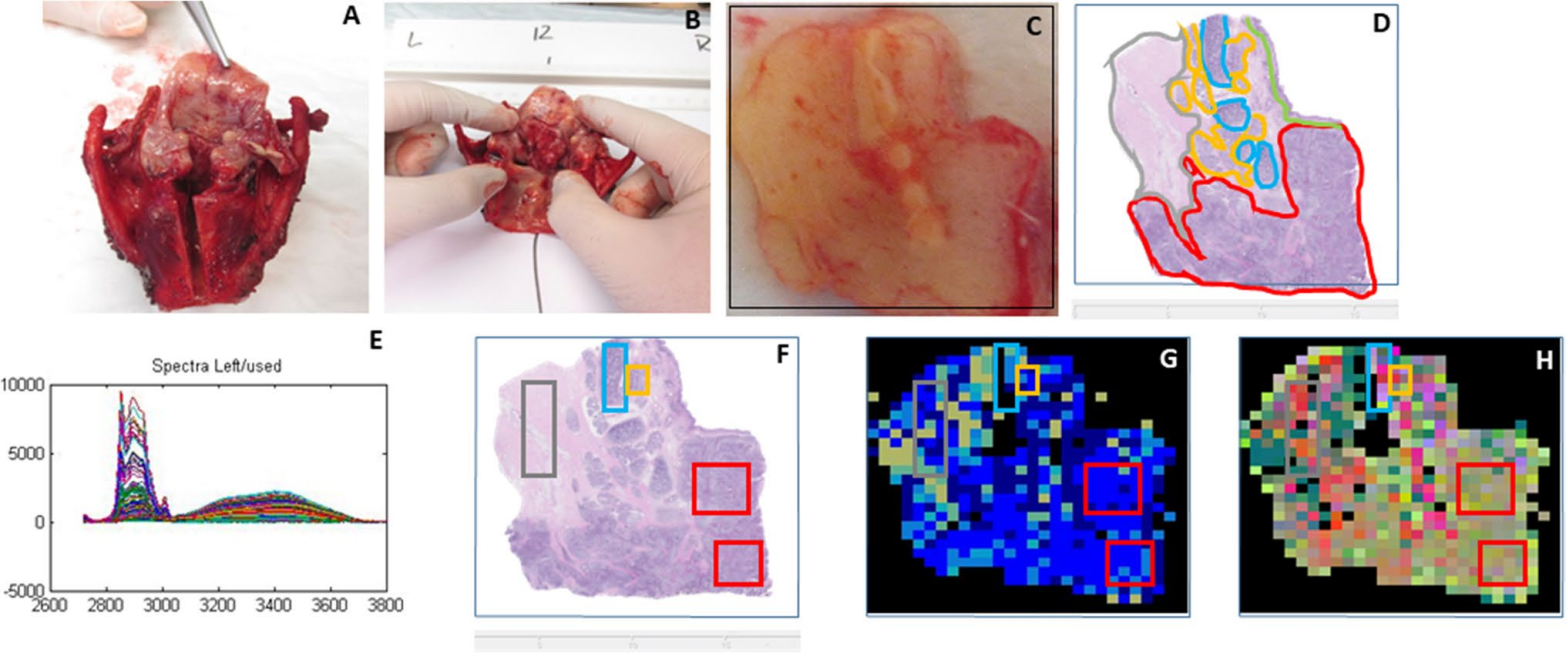
An overview of a single mapping experiment. **A** The resection specimen was cut open for intra-operative assessment of the resection margins. **B** A tissue section with tumor and non-cancerous surrounding tissue was obtained for the Raman experiment. **C** This Raman section was inserted into a cartridge and the whole area was measured. **D** The HE-stained Raman section with annotation of the different tissue types. **E** The Raman spectra measured for the Raman section. **F** Areas with unambiguous histopathology were selected for Raman analysis. **G** Raman water map with water distribution and projected histological annotation. Black pixels correspond to absence of tissue or to spectra with low Raman signal quality. **H** CH-stretching region PCA map and projected histological annotation. Black pixels correspond to absence of tissue or to spectra with low Raman signal quality

Figure 1 shows an overview of a single mapping experiment.

**Fig. 2:**
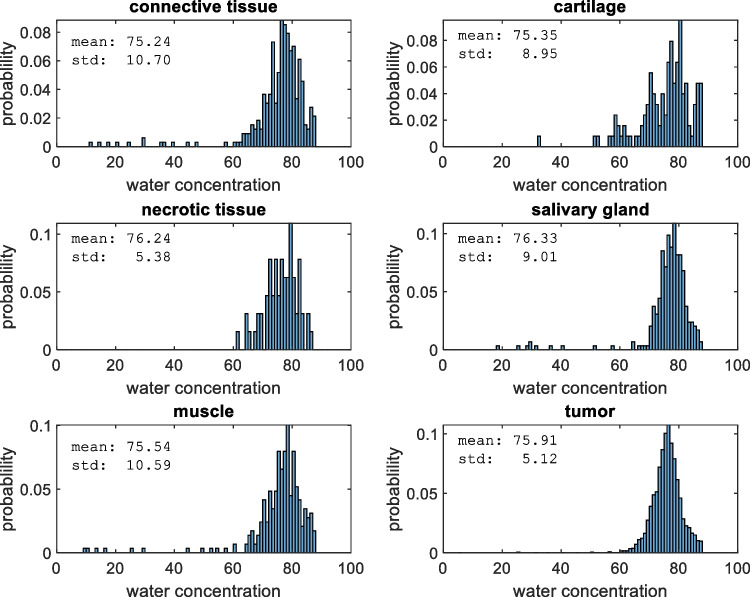
Histograms of the water concentration for the different tissue types

Figure 2 shows the histograms of the water concentration for the different tissue types with the mean and standard deviation (std) of the distribution. Although the water concentration distributions of the tumor and all different non-cancerous tissue structures are highly overlapping, the distributions of tumor and non-cancerous are significantly different (Wilcoxon’s rank sum test at the 0.05 confidence level: *p* <  < 0.01). Because of the high degree of overlap, the discriminative power of the classification is low (area under ROC curve: 0.56).

**Fig. 3:**
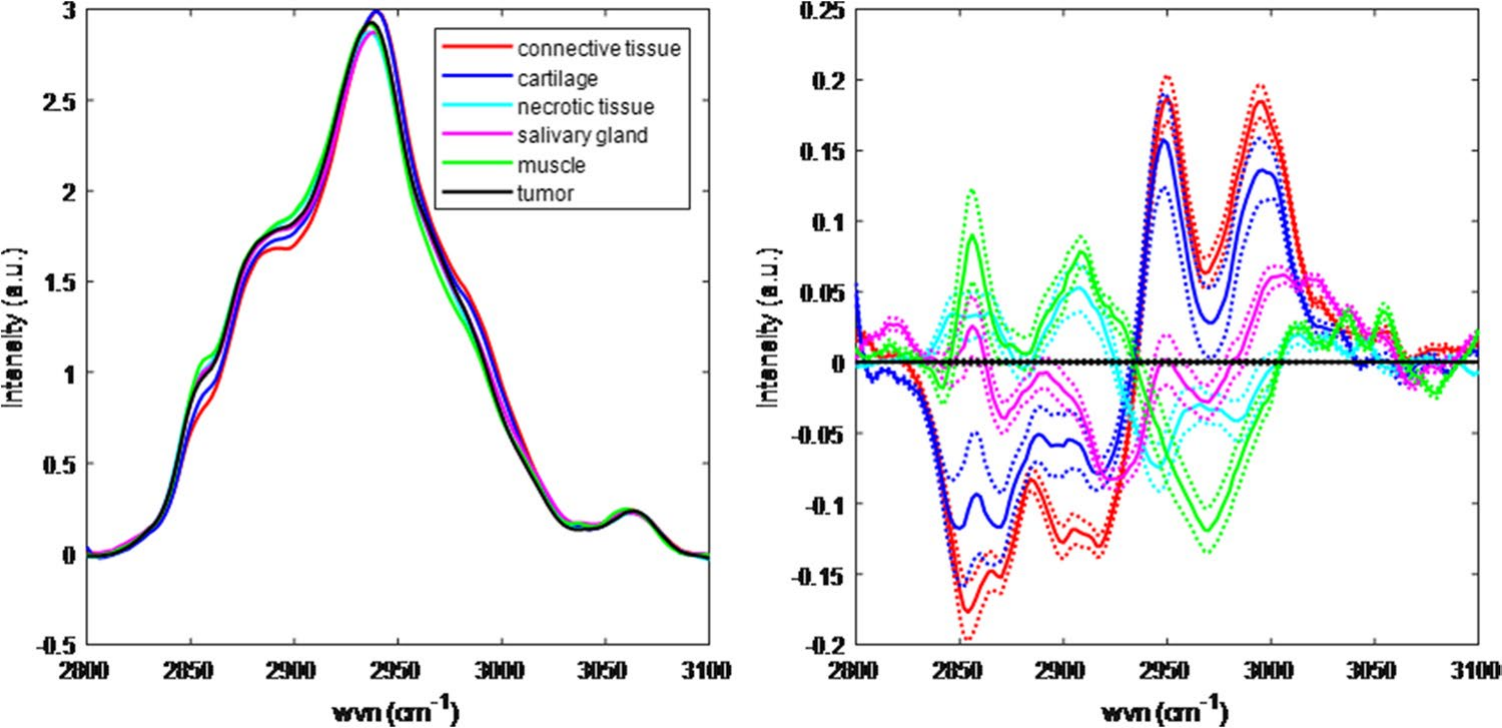
CH-stretching region of the spectra used for LDA. Left panel: mean spectra per tissue type. Right panel: the solid lines denote the differences with tumor for different tissue types and the dotted lines denote the ± standard error of the mean for the different tissue types

Considering the low discriminative power of the water concentration analysis, the CH-stretching region (2800–3100 cm^−1^) was investigated whether it contained more discriminative information. The spectra were EMSC-scaled and filtered as described in the “Material and methods” section. The left panel of Fig. 3 shows the mean spectra of the different tissue types in this region. The spectral differences between the mean spectra of tumor and of non-cancerous tissue types are shown in the right panel of Fig. 3.

**Fig. 4:**
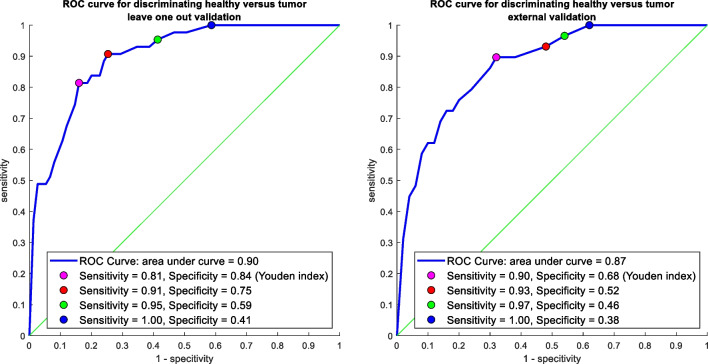
Left panel: ROC curve of leave-one-map-out validation on the model set; right panel: ROC curve of external validation using the independent data set

A PCA-LDA model was built using the model data set of 25 maps from 17 patients. The optimal number of input PCs for the PCA-LDA model was first determined by leave-one-map-out validation on the model data set. The discriminating power of the PC-LDA model (area under the ROC curve) was determined for different amounts of input PCs. The best number of input PCs, yielding the highest discriminating power (0.90), was found for a PCA-LDA model built on the scores of the first 4 PCs as input. The left panel of Fig. 4 shows the ROC curve of a leave-one-map-out validation of this PCA-LDA model. The PCA-LDA model was validated with an independent test data set of 9 maps from 5 patients. The right panel of Fig. 4 shows the ROC curve of the validation with the independent data set. As can be seen from the figure, the ROC curves for the model data set and the independent test data set give similar results, showing that there is consistent discrimination information present in the CH-stretching region of the Raman spectra of the larynx.

At the Youden index (optimal combination of sensitivity and specificity), the validation set showed a sensitivity of 0.90 and a specificity of 0.68. All false positives were analyzed to investigate which non-cancerous tissue structures are falsely identified as tumors. Forty percent of all necrotic tissue spectra were identified as tumor spectra, followed by the salivary gland (36%), connective tissue (18%), and cartilage (9%). Spectra from muscle were all identified as non-cancerous tissue spectra.

## Discussion

Therapeutic surgery guidance (i.e., facilitating the achievement of adequate resection margins) is an important clinical need during surgical oncological procedures. The aim of this study was to determine whether high wavenumber Raman spectroscopy can be used to discriminate laryngeal cancer from surrounding non-cancerous tissue.

Using the CH-stretching region only, it was able to classify tumors from non-cancerous tissue with a discriminative power of 0.87, validated on an independent dataset. Analysis of the false positive predictions shows that mostly necrotic tissue and salivary gland were identified as tumors. The mean spectra of these two tissue types also showed the smallest difference with the mean spectrum of tumors. The high false positive rate for the salivary gland is in line with earlier observations for oral cavity tissue where the salivary gland was also wrongly identified as a tumor in 35% of the cases [24]. The discriminative power is lower than the 0.97 reported by Lin et al. [16] in a study where they used a trans-nasal Raman spectroscopy technique integrated with an endoscope-based fiber-optic Raman probe to collect spectra of cancerous and non-cancerous laryngeal tissue in the CH-stretching region.

The results of this study show high water concentrations in both LSCC and all surrounding non-cancerous tissue structures. Despite the significant shape difference in the water concentration distributions for the tumor and surrounding non-cancerous tissue, the discriminative power of 0.56 is low. This confirms the results of Lin et al. [17] who reported a low diagnostic difference for the OH-stretching band around 3400 cm^−1^ to discriminate cancer from non-cancerous tissue in the larynx.

These findings are different from the results obtained in previous studies for the oral cavity, where consistently higher water concentration was found for OCSCC compared to surrounding non-cancerous tissue [19]. The water concentrations measured in non-cancerous laryngeal tissue are significantly higher than in non-cancerous oral cavity tissue.

Surprisingly, for all tissue types in the larynx, the mean water concentration is higher than in OCSCC. First, a literature search was performed to find an explanation for the higher water concentration such as physiological, anatomical, or molecular differences in the larynx compared to the oral cavity. Only for the vocal cords it has been shown that they have a high water concentration [27]. However, the vocal cords are a small part of the larynx, and no publication was found mentioning a generally higher water concentration in healthy laryngeal tissue compared to healthy oral cavity tissue.

Second, to confirm these surprising findings, water concentration measurements were performed on freshly excised tissue using a fiber-optic needle probe setup that was developed for the assessment of oral cavity cancer–based on water concentration. The same experiments were performed, as described above, with this new Raman setup for the larynx. The results from the Raman setup with the cartridge were confirmed with a high water concentration of ± 75% in all tissue types in the larynx. The details are described in the supporting material S1.

There are two limitations in the study design that may have contributed to the relatively low discriminative power of the classification model. The first limitation is related to the fact that to build a good classification model based on Raman spectroscopy, a large spectral database is needed with accurate histopathologic annotation. For the current study, it was only possible to retrieve small tissue sections from the resection specimen to not interfere with the standard pathological evaluation of the specimen. As explained by Author et al. [19], with a laser spot size of 4 µm, it is not always feasible to translate the exact position of the laser to the HE-stained section, resulting in only a limited number of spectra with an accurate annotation per tissue sample. The second limitation is the signal-to-noise ratio of the measurements. In this study, a signal acquisition time of 1 s was used. The selection of this acquisition time was based on earlier work. Due to the high water concentrations encountered, this was too short to obtain a sufficiently high signal-to-noise ratio in the CH-stretching region for optimal discrimination, given the small differences between tumor and non-cancerous tissue structures.

For this study, the high wavenumber region (2500–4000 cm^−1^) was chosen because of the stronger Raman signals, the reduced fluorescence background, and the easy in vivo implementation using a handheld Raman spectroscopy probe (i.e., single fiber probe without filters). Currently, biomedical Raman research in diagnosing cancer is mostly centered on the fingerprint region (i.e., 800–1800 cm^−1^) that contains rich biochemical information about the tissue. The advantage of the fingerprint Raman spectroscopy technique stems from its capability to uncover specific information about the backbone structures of proteins, lipids, and nucleic acid assemblies in cells and tissue. The fingerprint region has shown successful results for the differentiation of LSCC and non-cancerous tissue, with a 69–92% sensitivity and 90–94% specificity in previous studies [13–15]. Teh et al. [15] identified 21 Raman features related to the biochemical and biomolecular changes (e.g., proteins, lipids, nucleic acids, and carbohydrates) that are associated with LSCC. They developed a random forest algorithm and observed significant differences in Raman spectra between tumor and non-cancerous tissue with an overall accuracy of 89.3%, a sensitivity of 88.0%, and a specificity of 91.4% [15]. Some limitations are associated with the implementation of the Raman spectroscopy fingerprint region, especially if used in vivo. This region is hampered by the strong signal background generated by the optical fiber, requiring complicated probe designs with multiple fibers and filters which makes them expensive and difficult to reproduce [16–18]. Also, the signal intensity in the fingerprint region is relatively low with relatively large fluorescence backgrounds, which may cause long signal integration times making it impractical for clinical use [16–18].

Lin et al. [17] suggested that fingerprint and high wavenumber region Raman spectroscopy combined could have advantages for tissue characterization because of the complementary information. The diagnostic accuracy with integrated fingerprint and high wavenumber was found to be superior to either fingerprint (accuracy 86.1%) or high wavenumber (accuracy 84.2%) alone [17]. For clinical use, this would require a significant step in probe development that can be avoided if the use of the high wavenumber region alone provides sufficient clinical information.

Considering the current limitations and published studies, future work may include the exploration of a different setup that is optically designed to have an increased laser spot size (for instance, 250 µm) and spectral acquisition time. This can improve the histopathological correlation and increase the signal-to-noise ratio, and thus the discriminative power of the classification model. Deep learning algorithms can be incorporated for a more accurate classification model. However, for these deep learning models, the training data set must be extended to a larger cohort of patients, to better capture the variation in spectra, within and between patients.

## Conclusion

High wavenumber Raman spectroscopy can discriminate laryngeal cancer from non-cancerous tissue structures. Contrary to the findings for oral cavity cancer, water concentration is not a discriminating factor for laryngeal cancer. Despite the current limitations, this study contributes to important steps towards the development of a Raman spectroscopy probe for therapeutic surgery guidance.

### Supplementary Information

Below is the link to the electronic supplementary material.

Supplementary file1 (DOCX 3.08 MB)

## Data Availability

The data that support the findings of this study are available from the corresponding author upon reasonable request.
