# Supplementary material for: Raman spectroscopy to discriminate laryngeal squamous cell carcinoma from non-cancerous surrounding tissue
Source: Lasers Med Sci. 2023 Aug 25;38(1):193. doi: 10.1007/s10103-023-03849-4 (PMC10457228; doi:10.1007/s10103-023-03849-4)
Supplement: Supplementary file 1 — Supplementary file1 (DOCX 3.08 MB) [file 10103_2023_3849_MOESM1_ESM.docx]

# Supporting Material

## S1 – Raman experiments with a fiber-optic needle probe

### Materials and Methods

#### Fiber-optic needle probe prototype

A prototype Raman instrument, developed by the Erasmus MC, RiverD International B.V. The Netherlands, and art photonics GmbH. (Germany), was used (figure 1). The development was supported by the Dutch Cancer Foundation (project number: 8027 / KaWeFis Batch 5).

The instrument is comprised of a custom-designed high wave number Raman Module (RiverD), enabling signal collection in the spectral range of 2500-4000cm-1, a 671nm laser (Gem671, 50mW–250mW, LaserQuantum, UK), and a charge-coupled device (CCD) camera fitted with a back-illuminated deep depletion CCD-chip (Andor iVac, 316 LDC-DD, Andor Technology Ltd., UK).

The Raman module is connected to the fiber-optic needle probe through a cable (figure 1.a) containing a fiber-optic patch-cord (NIR 100/110AL-300-FC/PC-MS44, art photonics GmbH., Germany) and electrical wiring.

The fiber-optic needle probe is disposable and consists of a fiber-optic needle (30G x 12 mm Omnican® insulin needle, B. Braun Holding GmbH & Co., Germany) that has a 100µm core fiber-optic inside (NIR100/110AL, AP11396, art photonics GmbH., Germany) (figure 1.b). It is attached to an actuator (via FC/PC) that drives the needle into the tissue to a maximum depth of 10mm (SmartAct type SLC1730L Linear positioner, SmarAct GmbH., Germany). The actuator is located inside the probe's plastic housing, to which the patch cable is attached (figure 1.b).


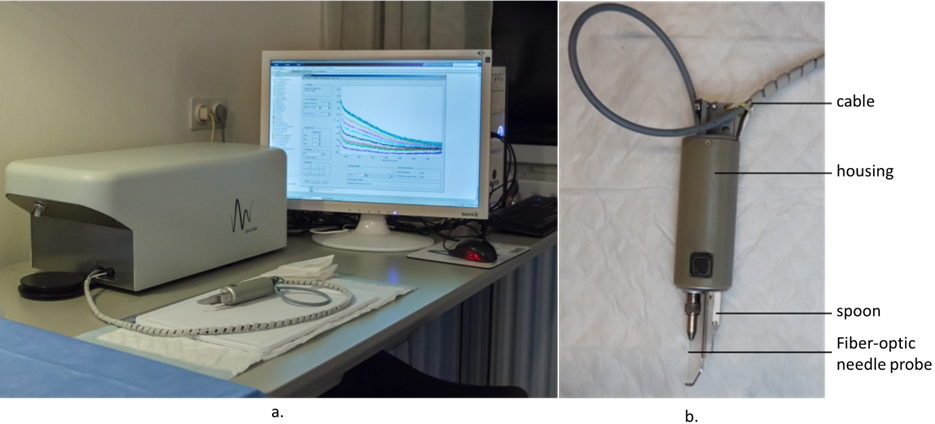
The spectral resolution of the fiber-optic needle probe prototype is >15cm-1. The depth sensitivity is approximately 100μm. This was experimentally determined based on the full half-width maximum of the step response (measured in a non-scattering medium).

*Figure 1 Fiber-optic needle probe prototype. a. Complete system. b. Close-up of the fiber-optic needle probe*

#### Tissue sampling and data collection


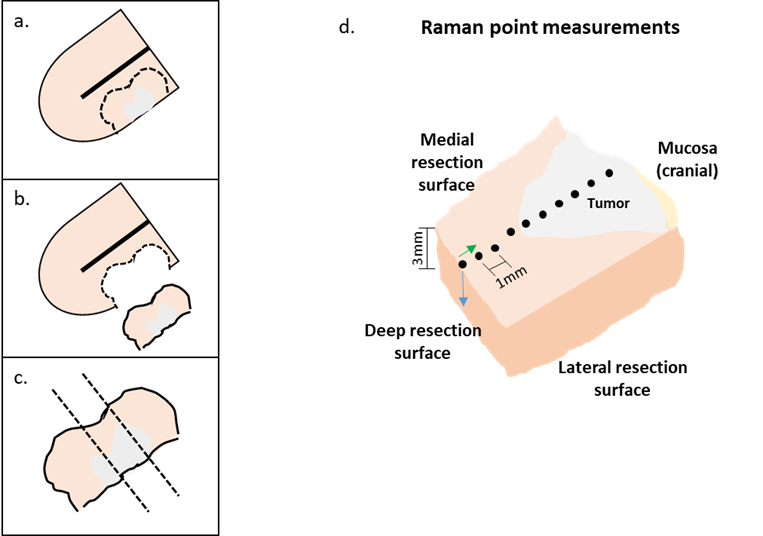
A Raman section was retrieved as described in the materials and methods of the main article. Raman point measurements were performed with the fiber-optic needle probe prototype from the surface of the Raman section to a depth of ≤3mm with a step size of 0.5mm (figure S2, blue arrow). Per step, a Raman spectrum was collected with an exposure time of 0.1s and an average laser power of ≈80mW (at the fiber-optic needle probe tip). Each point measurement was 1mm distant from the next measurement (figure S2, green arrow).

*Figure 2 Raman section with point measurements from the resection surface (black dots). Each point measurement is at 1mm distance from the next (green arrow). Per point measurement, the fiber-optic needle probe collects Raman spectra from the surface to a depth of* ≤3*mm and with a step size of 0.5mm (blue arrow). Per step, one Raman spectrum is collected with an exposure time of 0.1s.*


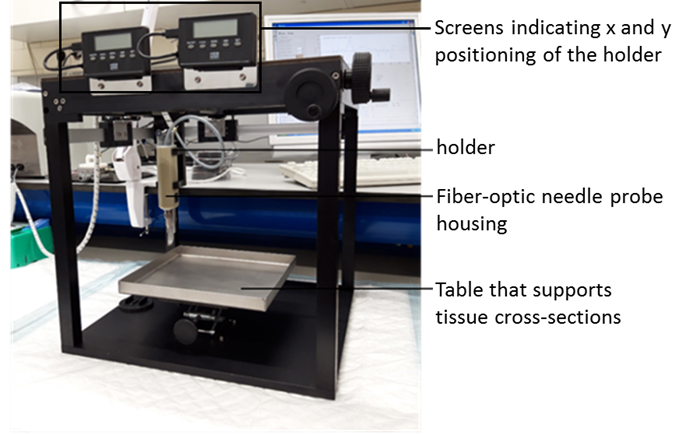
To allow the control of x and y positioning of the probe, a module for semi-automated scanning of the surface was used. This module contains a holder that grips on the probe housing (figure 3).

*Figure 3 Module for semi-automated scanning of the surface of the Raman section*

After the experiment, the Raman section was added to the main specimen for fixation in formalin, for routine histopathologic evaluation.

#### Histopathology

Histopathologic evaluation of the Raman section was performed as described in the main article.

#### Calibration and pre-processing of spectra

All spectra were corrected for the instrument's wavelength-dependent detection efficiency and calibrated according to the instructions of the spectrometer supplier on the relative wavenumber axis (RiverD). Cosmic ray events were removed, and the signal background generated in the setup's optical path was subtracted [1]. MATLAB (Mathworks, Natick, MA, USA) was used for data processing and data visualization. Saturated spectra and spectra with low signal intensity were discarded (less than 5% of the average spectra signal intensity). The intensity was measured within the range 2,700cm-1 and 3,800cm-1. Low signal intensities were found at positions where the probe tip was not in full contact with the tissue. Fluorescence background signal was determined and subtracted using the multiple regression fitting method developed by *Barroso et al.* [2].

#### Spectral analysis

The analysis of the spectra was performed in MATLAB. Spectral analysis consisted of the extraction of Raman features that are strong discriminators of laryngeal squamous cell carcinoma (LSCC) and non-cancerous tissue.

##### Water content

The water content was extracted by calculating the ratio of the signal intensity between the bands at 3390cm-1 and 2935cm-1 for each calibrated and background corrected spectrum according to the method developed by *Caspers et al.* (2001) and described in detail in previous studies [3]. A comparison between the frequency distribution of the water content for tumor spectra and for non-cancerous tissue spectra was performed.

### Results


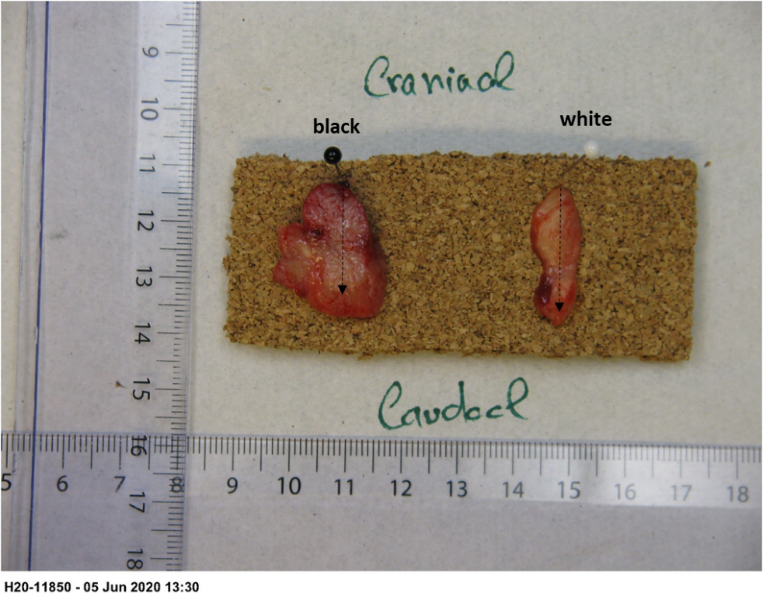
Forty Raman point measurements were performed on two Raman sections from 1 laryngectomy specimen (figure 4). A pin was used to mark the first Raman point measurement, closest to the resection surface.

Figure 4 Raman sections: measurements with the needle probe were performed in a line, starting at the pin


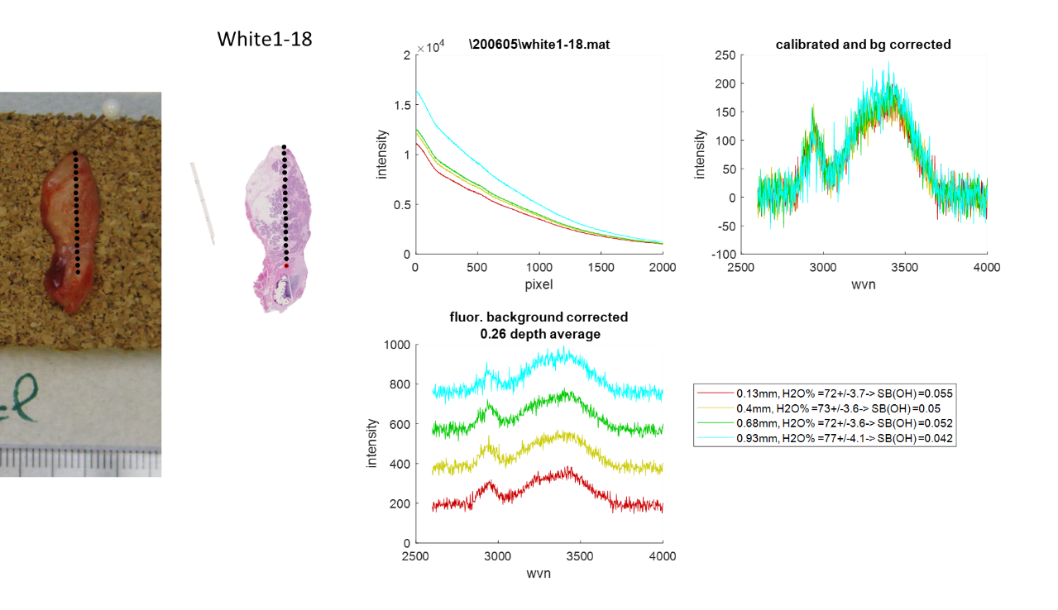
An example of a point measurement in non-cancerous tissue is shown in figure 5 and an example for tumor is shown in figure 6.

*Figure 5 Example of a measurement on non-cancerous laryngeal tissue*


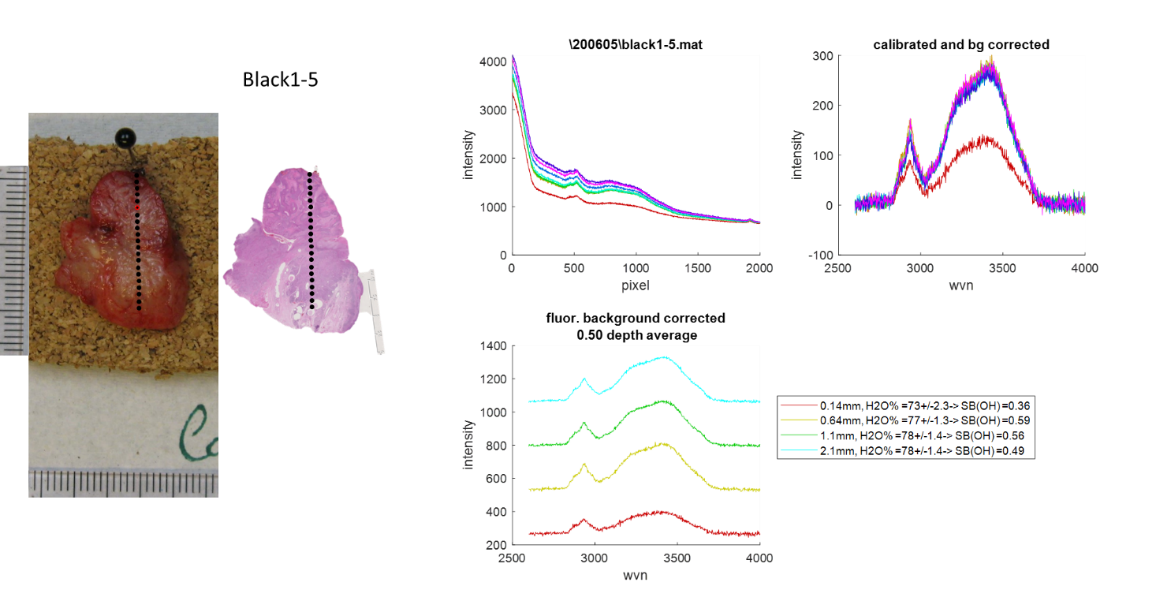
Figure 6 Example of a measurement on LSCC


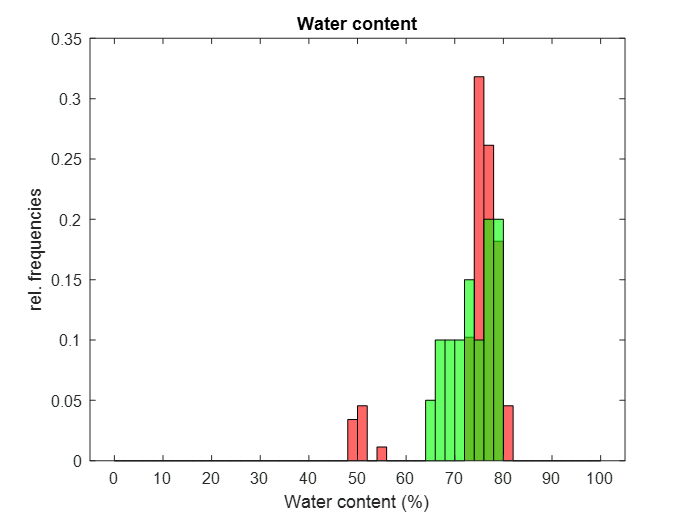
The histogram of relative frequencies of water content distribution for non-cancerous tissue and tumor is shown in figure 7. It shows that the water concentrations of non-cancerous tissue are high and highly overlapping with the water concentrations of tumor.

Figure 7 Water concentration distribution for laryngeal non-cancerous tissue (green) and laryngeal tumor (red)

## References

1. Wolthuis R, B. S. T., Caspers PJ,BuschmanHPJ, R€omerTJ, Bruining HA. (1999). "Raman spectroscopic methods for in vitro and in vivo tissue characterization." Fluorescent Luminescent Probes Biol Activity: 433–455.

2. Barroso, E. M., Ten Hove, I., Bakker Schut, T. C., Mast, H., van Lanschot, C. G. F., Smits, R. W. H., Caspers, P. J., Verdijk, R., Noordhoek Hegt, V., Baatenburg de Jong, R. J., Wolvius, E. B., Puppels, G. J., & Koljenović, S. (2018). Raman spectroscopy for assessment of bone resection margins in mandibulectomy for oral cavity squamous cell carcinoma. European journal of cancer (Oxford, England : 1990), 92, 77–87. <https://doi.org/10.1016/j.ejca.2018.01.068>

3. Caspers, P. J., Lucassen, G. W., Carter, E. A., Bruining, H. A., & Puppels, G. J. (2001). In vivo confocal Raman microspectroscopy of the skin: noninvasive determination of molecular concentration profiles. The Journal of investigative dermatology, 116(3), 434–442. https://doi.org/10.1046/j.1523-1747.2001.01258.x
